# The Hsc70 disaggregation machinery removes monomer units directly from α-synuclein fibril ends

**DOI:** 10.1038/s41467-021-25966-w

**Published:** 2021-10-14

**Authors:** Matthias M. Schneider, Saurabh Gautam, Therese W. Herling, Ewa Andrzejewska, Georg Krainer, Alyssa M. Miller, Victoria A. Trinkaus, Quentin A. E. Peter, Francesco Simone Ruggeri, Michele Vendruscolo, Andreas Bracher, Christopher M. Dobson, F. Ulrich Hartl, Tuomas P. J. Knowles

**Affiliations:** 1 Yusuf Hamied Department of Chemistry, Centre for Misfolding Diseases, University of Cambridge, Lensfield Road, Cambridge, CB2 1EW UK; 2 Department of Cellular Biochemistry, Max-Planck Institute of Biochemistry, Am Klopferspitz 18, 82152 Martinsried, Germany; 3 Munich Cluster for Systems Neurology (SyNergy), Munich, Germany; 4 Department of Physics, Cavendish Laboratory, University of Cambridge, JJ Thomson Road, Cambridge, CB3 0HE UK; 5 Present Address: ViraTherapeutics GmbH, 6063 Rum, Austria

**Keywords:** Chaperones, Protein aggregation, Kinetics, Single-molecule biophysics, Biophysical chemistry

## Abstract

Molecular chaperones contribute to the maintenance of cellular protein homoeostasis through assisting de novo protein folding and preventing amyloid formation. Chaperones of the Hsp70 family can further disaggregate otherwise irreversible aggregate species such as α-synuclein fibrils, which accumulate in Parkinson’s disease. However, the mechanisms and kinetics of this key functionality are only partially understood. Here, we combine microfluidic measurements with chemical kinetics to study α-synuclein disaggregation. We show that Hsc70 together with its co-chaperones DnaJB1 and Apg2 can completely reverse α-synuclein aggregation back to its soluble monomeric state. This reaction proceeds through first-order kinetics where monomer units are removed directly from the fibril ends with little contribution from intermediate fibril fragmentation steps. These findings extend our mechanistic understanding of the role of chaperones in the suppression of amyloid proliferation and in aggregate clearance, and inform on possibilities and limitations of this strategy in the development of therapeutics against synucleinopathies.

## Abstract (web summary)

Molecular chaperones from the Hsp70 family can break up protein aggregates, including amyloids. Here, the authors utilize microfluidic diffusional sizing to assess the mechanism of α-synuclein (αS) disaggregation by the Hsc70–DnaJB1–Apg2 system, and show that single αS molecules are removed directly from the fibril ends.

## Introduction

Misfolding and aggregation of proteins and peptides into amyloidogenic fibrils are hallmarks of a wide range of neurodegenerative disorders^1–3^, including α-synuclein (αS) in Parkinson’s disease, the Aβ-peptide in Alzheimer’s disease, and Huntingtin (HTT) in Huntington’s disease^4^. The accumulation of such fibrillar deposits in the central nervous system occurs in an age-dependent manner; earlier in life, this process is counteracted by efficient cellular protein quality control machinery that inhibits the amyloid formation and thus disease^5–7^.

Molecular chaperones are critical components of this quality control system^7^. Initially identified as part of the heat stress response^8–10^, chaperones have been shown to assist protein folding and rescue misfolded states^6,8^. Particularly variants of the 70 kDa heat shock protein family (Hsp70s) populate some of the most critical nodes in the proteostasis network and are involved in assisting protein folding and in exerting holdase activity. They are also actively engaged in preventing protein aggregation and in degrading misfolded proteins, as well as in mediating the assembly and disassembly of oligomeric protein species^7,11,12^. In fact, Hsp70s, along with various other chaperones, have been shown to modulate essentially all microscopic steps in amyloid formation, including elongation^13,14^, primary nucleation^15^, as well as secondary nucleation^16^.

In recent years, mounting evidence has indicated that Hsp70 chaperones are also involved in the disassembly of aggregates and are capable of disaggregating even persistent amyloidogenic aggregate structures such as αS, HTT and Tau fibrils^17–27^. In particular, the constitutively expressed chaperone heat shock cognate Hsc70 (HSPA8) together with the Hsp40 class B J-protein DnaJB1 and the Hsp110 family nucleotide exchange factors (NEFs) Apg2 or Hsp105α have been demonstrated to constitute a powerful ATP-driven disaggregase system that disassembles amyloids within minutes, promoting their fragmentation and depolymerisation into monomers or smaller oligomeric structures^17,18,20^.

**Fig. 1:**
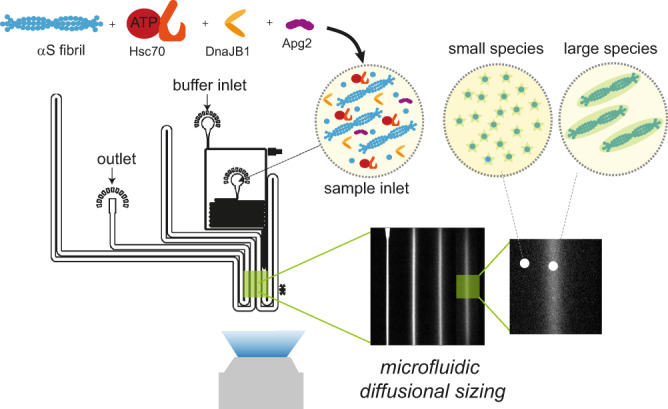
Hsc70-mediated αS fibril disaggregation monitored by direct size measurements through microfluidic diffusional sizing. αS fibrils are incubated with the Hsc70–DnaJB1–Apg2 chaperone triad. At different time points, the reaction mixture is analysed using microfluidic diffusional sizing which probes the sizes and hence molecular weights of the species present in the solution. From the recorded diffusion profiles, the size decay of fibrils with time is monitored, allowing kinetic and mechanistic analysis of the disaggregation reaction.

Several studies have provided substantial insights into the basic working principles of the Hsc70–DnaJB1–Hsp110 triad chaperone system (Fig. 1)^7,18,19,28^. Structurally, Hsc70, as other Hsp70 chaperones, contains an N-terminal nucleotide-binding domain (NBD) of 40-kDa, which is linked via a flexible, hydrophobic linker to a 15-kDa substrate-binding domain (SBD) and a 10-kDa α-helical lid^7,11,12^. The SBD recognises hydrophobic peptide segments that are exposed in non-native substrate proteins^29–31^, which are delivered to Hsp70 by J-protein chaperones such as DnaJB1. Upon ATP hydrolysis to ADP induced by the J-protein, the lid closes to form a stable substrate–Hsp70 complex and the J-protein dissociates from Hsp70^7,11,12^. NEFs, in particular members of the Hsp110 family (e.g., Apg2 or Hsp105α)^32–36^, replace the bound ADP with ATP, facilitating lid-opening and substrate release. Since assembly of the chaperone machinery on a protein aggregate leads to a significant entropy loss due to excluded volume effects, the chaperone is thought to act on its substrate by entropic pulling, that is, by exerting a force of up to 15–20 pN to the region it is bound to, leading to the fibril disaggregation^37–39^.

While basic aspects of the functional cooperation of the Hsc70–DnaJB1–Apg2 chaperone system in protein disaggregation have been established,^7,18,19^ key mechanistic questions remain unanswered. Specifically, it is unclear if the disaggregase machinery acts predominantly on the fibril ends or along the fibril surface, and if the monomer is removed from the fibril substrate, or whether smaller fragments are produced. Moreover, fundamental functional and biophysical parameters such as binding stoichiometry and binding affinities describing the interaction between the participating components are yet to be resolved. Insights into these aspects are of fundamental importance, not least because of the therapeutic potential of Hsp70-mediated disaggregation in neurodegeneration.

Here, we dissect the molecular mechanisms by which the Hsc70–DnaJB1–Apg2 chaperone system disaggregates αS fibrils. We employ microfluidic diffusional sizing in conjunction with chemical kinetics analysis to quantify and characterise the molecular species formed during disaggregation (Fig. 1). A key finding of this study is that the chaperones disassemble αS fibrils into monomers. This ATP-dependent process requires all three components of the Hsp70 system and follows pseudo-first-order kinetics, which suggests that monomer units are removed directly from the fibril ends. Indeed, single-round disaggregation experiments clearly show that αS monomer is produced after a single disaggregation cycle in a step mediated by the action of Apg2 on fibril-bound Hsc70. Lastly, we assess the binding properties between the different chaperones and co-chaperones as well as between the chaperones and the fibrils. Based on these results, we establish a full kinetic and thermodynamic profile of the Hsc70-mediated disaggregation reaction and, consistent with recent findings^19^, propose a model of αS disaggregation suggesting that Hsc70 chaperones form a cluster in order to exhibit disaggregase functionality on αS fibrils.

## Results

### Kinetics of αS fibril disaggregation by the Hsc70 chaperone machinery

**Fig. 2:**
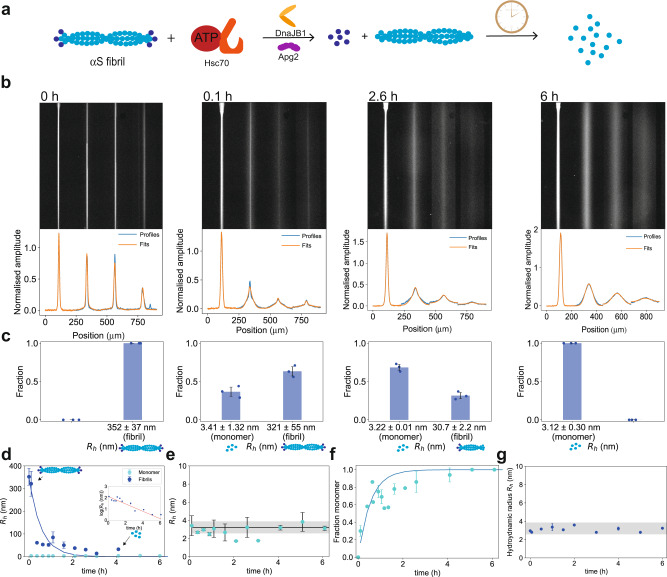
Diffusional sizing and kinetic analysis of αS fibril disaggregation by the Hsc70 chaperone machinery. **a** Schematic of the disaggregation reaction and the experimental assay. **b** Representative images of microfluidic diffusional sizing experiments at different time points and diffusion profiles obtained from image analysis during the disaggregation time course. The four different channels represent four different diffusion times; the less diffusive spreading there is in these four channels, the larger the molecule is. It can be seen that diffusion profiles broaden over time, indicating that a smaller species is created during the disaggregation reaction. At 0.1 h and 2.6 h, the profiles show an overlay of two species, one with high diffusivity, one with little diffusivity, indicating a heterogeneous population of a small, broadly diffused-out species and a larger, little diffusing species was observed, while the population was homogeneous at 0 h and 6 h. Extracted *R*_h_ values for the small and large species from diffusion profile fitting at these time points are reported in panel **b**. The experimentally obtained diffusional profiles are shown in blue and the obtained fits in orange. **c** Histograms showing the size distribution of the two species from image analysis. At 0 h and 6 h, *R*_h_ values of single-component fits are reported. The individual data points are overlayed on the bar plot. **d** Evolution of *R*_h_ for small (cyan) and large species (blue) over time. The size of the larger species decayed monotonically, consistent with a single exponential fit (blue line). The size of the smaller species remained constant over time. Inset: logarithmic representation of *R*_h,large_ over time. From these fits, a rate constant of *k* = 1.8 ± 0.3 × 10^–4^ s^–1^ was determined (*R*^2^ = 0.89). **e** *R*_h_ of the small species compared to *R*_h_ of pure monomer (black line and grey region indicate the expected size and size range from pure monomer). This analysis shows that monomer is produced throughout the reaction and no other intermediate is generated. **f** Fraction of monomer over time (cyan), showing that the fraction of monomer increases over time, whereby less monomer is generated at later time points, consistent with Fig. 2d. (*R*^2^ = 0.89). **g** Time course experiment. 100 nM αS monomers labelled with Alexa Fluor 488 are added to a disaggregation mixture with 2 μM unlabelled αS fibrils, and the size of the fluorescent species is measured over time. The size remains largely constant over the time course of the 6 h experiment, showing the inhibition of elongation through the disaggregation machinery. Data in **c**–**g** are represented as mean ± standard deviation of *n* = 3 independent experiments.

To gain insights into the mechanism of αS fibril disaggregation, we sought to obtain quantitative data on the time evolution of this process directly in solution. To this effect, we applied a microfluidic diffusional sizing approach^40–42^ to investigate the time evolution of αS fibril disaggregation by the Hsc70 chaperone machinery. Such heterogeneous multi-component systems can be challenging to study using conventional surface-based analysis approaches, but the absence of convective mixing on microfluidic scales allows these interactions to be investigated directly in solution without the requirement for any of the binding partners to be immobilised onto a surface. Alternative methods such as dynamic light scatting (DLS) have been used to analyse heterogeneous aggregation reactions^43–46^, but can be challenging to apply to multi-component systems like the one studied here where the specific signal originating from the chaperone proteins would obscure the signal of αS disaggregation products. The diffusional sizing approach overcomes this problem by measuring the diffusivity of fluorescently labelled molecular species in solution and monitoring changes as they undergo binding events. In practice, we capture the diffusion process in both space and time by acquiring the longitudinal diffusion profiles of protein molecules, here αS species, flowing in a microfluidic channel (Fig. 2a). The diffusion profiles are then analysed by considering advection–diffusion processes to extract the distribution of diffusion coefficients (not shape-dependent) and the corresponding hydrodynamic radii (*R*_h_) of the individual species present in solution^40,42^. The diffusion coefficient is, according to the Stokes–Einstein relationship, inversely proportional to the hydrodynamic radius^40^. Therefore, increased broadening of the diffusion profile is expected for smaller proteins in comparison to larger proteins. Crucially, this method also allows for the detection of protein–protein interactions by monitoring the increase in size of diffusing species associated with binding^16,47^. Thus, microfluidic diffusional sizing allows distinguishing different species in αS fibril disaggregation based on their hydrodynamic radii.

In the first series of experiments, we monitored the kinetics of αS fibril disaggregation by the Hsc70 disaggregation machinery. To this end, we incubated the chaperone system consisting of Hsc70, DnaJB1, and Apg2 in presence of an ATP-regenerating system (see the “Methods” section and Fig. 2a) with preformed αS fibrils labelled with AlexaFluor488 (amine labelling of αS monomers). These fibrils were produced by mixing 10% labelled and 90% unlabelled monomers. Such labelled fibrils were structurally comparable to unlabelled fibrils, as shown by atomic force microscopy (AFM) (Supplementary Fig. 1) and were disaggregated with similar kinetics as unlabelled fibrils in bulk assays followed by thioflavin T (ThT) fluorescence (Supplementary Fig. 2). The sample was introduced at the centre of the microfluidic channel and the extent of diffusion towards the edges of the channel was monitored as a function of channel position. A solution of pure fibrils in the absence of the disaggregation machinery consisted of a high molecular weight species with the only little broadening of the sample stream along the microfluidic channel (Fig. 2b, 0 h), in agreement with the expected large size of αS fibrils (~200–300 nm, Supplementary Fig. 3). By contrast, a clear signature of fibril disintegration became apparent in the presence of the disaggregase system as diffusion profiles broadened over the time course of the reaction.

At early time points (Fig. 2b, 0.1 h), fibrils were still predominant; however, a second protein species with a high degree of diffusive broadening became apparent, showing that the initiation of the disaggregation reaction is fast (i.e., within a few minutes) and that a smaller molecular weight species is being produced. At intermediate time points (Fig. 2b, 2.6 h), the fluorescence intensity arising from the smaller diffused-out species increased relative to the larger species, while fibrils were still present. At late time points (Fig. 2b, 6 h), the diffusion profiles broadened significantly and yielded a monodisperse population of the smaller molecular weight species. Full disaggregation was observed after ~3 h. Crucially, disaggregation required the presence of all three chaperone components and ATP. Specifically, Hsc70 alone with ATP was unable to mediate fibril disaggregation (Supplementary Fig. 4).

Quantitative analysis of the diffusion profiles as a function of time revealed that the profiles are best described by two diffusing species (Fig. 2c and Supplementary Fig. 5). The fraction of the larger species with an initial *R*_h_ of ~350 nm, corresponding to the size of fibrils (Fig. 2d), decayed monotonically over time, while simultaneously the fraction of a smaller, diffused-out species with an *R*_h_ of ~3 nm gradually increased (Fig. 2e, f). The smaller species, that built up in time, corresponds in size to pure monomer (Fig. 2e), demonstrating that the reconstituted Hsc70 machinery disaggregates αS fibrils into monomer units. As shown in Supplementary Fig. 6, we are able to distinguish fluorescently labelled monomers from fibrils, oligomers from fibrils, and monomers from oligomers and to quantify the ratio of the present species accurately. In contrast, as shown in Supplementary Fig. 7, in DLS measurements it is challenging to obtain this level of resolution in a heterogeneous mixture and the method largely reports on the larger species present in solution under these conditions.

The observation that monomer was already abundant at very early time points not only highlights the fact that the fibril-to-monomer conversion reaction is very fast (i.e., on the minutes timescale) but also strongly indicates that monomer units are removed from the fibril ends directly, as opposed to scenarios that involve intermediate fragmentation steps, in which case other species than fibrils and monomer would have been observed. No monomer is observed in the fibril-only sample (Fig. 2b, 0 min).

Further quantification revealed that the size decay of the large fibrillar species was best described with a single exponential kinetic model, yielding a rate constant of *k* = 1.8 ± 0.3 × 10^–4^ s^–1^. Crucially, the apparent size of the species converged to that of the smaller species (i.e., monomer) after ~3 h, indicating that the disaggregation reaction had gone to completion. The single exponential behaviour was observed independent of whether the experiments were performed with full-length fibrils (see above) or sonicated fibrils (Supplementary Fig. 8). However, the reaction rate per synuclein concentration for the complete conversion of fibrils to monomer was accelerated by ~20% for the sonicated fibrils, which are ~20% smaller (270 vs. 350 nm), indicating that the abundance of more fibril ends allows a faster decline, as the Hsc70 is present in excess under the disaggregation reaction conditions (Supplementary Fig. 9). This finding supports the conclusion that the removal of monomer, simultaneously from both the fibril ends, depends on the number of fibril ends. A single exponential behaviour is consistent with a pseudo-first-order kinetic model (Fig. 2d) and thus supportive of a one-step disaggregation reaction mechanism, without previous fragmentation of the fibril.

Lastly, we investigated whether the disaggregation machinery also has the capacity to inhibit the forward aggregation reaction. To this effect, we performed an experiment where we added labelled monomer to unlabelled fibrils in the presence of the chaperone system, without detecting an increase in size (Fig. 2g), as opposed to the absence of chaperone (Supplementary Fig. 10). This suggests that the presence of chaperones in the aggregation mixture prevents monomer re-binding and, thus, fibril elongation. This mechanism is complementary to the active dissociation capacity of the chaperone machinery and means that freshly dissociated monomer units are protected against immediately being reincorporated into aggregates, thus enhancing the overall efficiency of the disaggregation process.

**Fig. 3:**
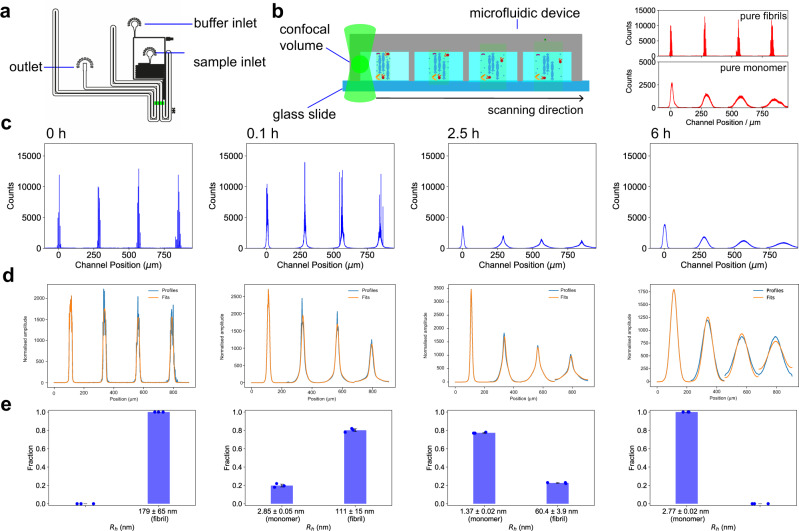
Analysis of αS fibril disaggregation by the Hsc70 chaperone machinery using confocal microfluidic diffusional sizing. **a** Design of the microfluidic device for confocal microfluidic diffusional sizing. The chip design is the same as for the epifluorescence microscopy experiments (see Fig. 1). **b** The confocal volume scans across the four innermost channels of the microfluidic chip made of polydimethylsiloxane (PDMS) (highlighted in panel **a**), thereby capturing the diffusive broadening of the reaction mixture with increased diffusion time. (Right) Typical diffusion profiles for pure fibrils and monomers. Pure fibrils show large fluorescence bursts due to the high number of fluorophores detected per fibril, as well as little broadening due to the large size of the fibrils. Profiles for pure monomer samples exhibit no bursts, due to the bulk concentrations employed and because each detected monomer only carries one label. Monomer profiles are broadened significantly in comparison to fibrils due to the small hydrodynamic radius of the monomeric protein. **c** Diffusion profiles for confocal scanning across the microfluidic channel for αS fibrils at different time points during the disaggregation reaction. Consistent with Fig. 2, the width of the profile base broadens significantly. Furthermore, the bursts, indicating the presence of numerous fluorophores as found in a fibrillar state, vanish at later time points, consistent with the disappearance of fibrils as a result of Hsc70-mediated disaggregation. **d** Fitting generated for profiles in panel (**c**). Normalised experimentally obtained diffusional profiles are shown in blue and the obtained fits in orange. **e** Histograms showing the size and fractional distribution of the two species from diffusion profile analysis. At 0 and 360 min, *R*_h_ values of single-component fits are reported. The individual data points are overlaid on the bar plot. Data in **e** are represented as mean ± standard deviation of *n* = 3 independent experiments.

To further explore the dissociation mechanism, we monitored the outcome of the disaggregation reaction using diffusional sizing combined with confocal microscopy^48–50^ (Fig. 3a, b). In this approach, the molecular diffusivity of sample components is probed by moving the confocal observation volume across the microfluidic device at the mid-height of the channel perpendicular to the flow direction, as shown in Fig. 3b (left panel). By measuring the fluorescence signal of molecules passing the confocal volume along the scan trajectory, the number of molecular species of different diffusion coefficients and hence different sizes can be measured directly.

We first performed experiments on pure fibrils and pure monomers in the absence of the disaggregation machinery to establish the signature of the two species present in disaggregation reactions (Fig. 3b, right panel). For fibrils, large fluorescent bursts located at the centre of the channel, corresponding to the detection of single aggregates, were observed. Fibrils contain a large number of fluorophores since 10% of the monomers within the fibril are fluorescently labelled. This results in the detection of burst events of high fluorescence intensity that are narrowly distributed around the centre of the channels and indicate a low diffusion coefficient and correspondingly large size. For the monomer sample, which was probed at nanomolar concentrations, the sizing profiles exhibited a broader spread, due to the larger diffusivity of the monomeric units as compared to fibrils. The signal is continuous because nanomolar concentrations are used, and therefore multiple monomeric units traverse the confocal detection volume at the same time, resulting in a bulk fluorescence signal rather than individual single-molecule events, in contrast to the aggregate sample.

After having established the properties of fibrillar and monomeric samples, we monitored the kinetics of Hsc70-mediated αS fibril disaggregation using confocal microfluidic diffusional sizing. At time point zero (Fig. 3c, d, 0 h), again narrowly distributed profiles with large bursts were recorded, consistent with fibrillar species. Shortly after starting the disaggregation reaction (Fig. 3c, 0.1 h), profiles started to broaden at the profile base, with bursts still being detectable. This indicates the emergence of a second, small protein species with a high degree of diffusive broadening. At intermediate time points (Fig. 3c, 2.5 h), the fluorescence intensity arising from the smaller diffused-out species increased relative to the larger burst species. At late time points (Fig. 3c, 6 h), the diffusion profiles broadened significantly and yielded a monodisperse population of the smaller molecular weight species. No large burst signals that would stem from fibrils were detected at this late time point. Diffused out species of the diffusion profiles of the disaggregation reaction were identical to the profiles obtained from the pure monomer sample. Quantitative analysis of the diffusion profiles of all time points during the disaggregation reaction using a two-species fitting procedure (Fig. 3d, e) yielded sizes and fractional distributions consistent with fibrils and monomer, in line with the results obtained from widefield diffusional sizing measurements (cf., Fig. 2). The analysis was performed with the same two species fitting as mentioned before for the widefield diffusional sizing measurements and as detailed in the “Methods” section.

### Single round disaggregation experiments

**Fig. 4:**
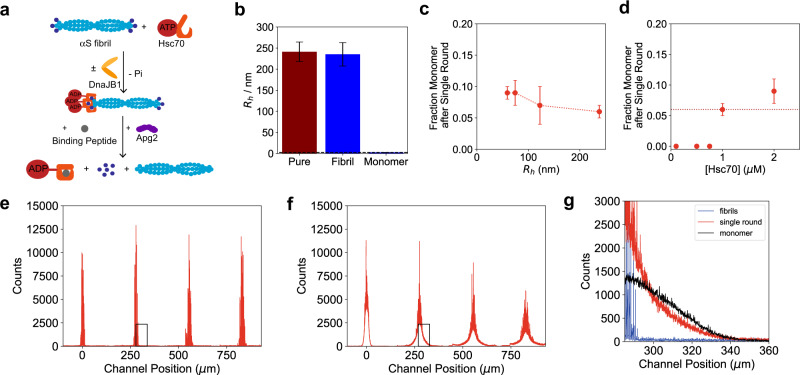
Single round disaggregation experiments. **a** To measure the outcome of a single disaggregation round, αS fibrils (2 µM monomer equivalent) were incubated with Hsc70 and DnaJB1. Subsequently, a 200-fold molar excess of Hsc70-binding peptide GSGNRLLLTG was added, to block Hsc70 re-binding to fibrils. **b** Outcome of a single disaggregation round, as measured by epifluorescence detection. Two species were detectable, the larger of which had a similar size to the initial fibril, the second of which had the size of monomer. Red represents pure fibrils, blue the outcome after a single disaggregation round. **c** Fraction of monomer generated as a function of fibril length at constant αS concentration, corresponding to an increased number of fibril ends. Shorter fibrils lead to more monomer release per single disaggregation round than longer fibrils, whereby this effect plateaus for fibrils longer than 100 nm. **d** Fraction of monomer measured at different Hsc70 concentrations. No disaggregation took place for Hsc70 concentrations below 1 µM, suggesting that there is a critical Hsc70 concentration required for efficient disaggregation. Data in **b**–**d** are represented as mean ± standard deviation of *n* = 3 independent experiments. **e** Diffusion profile for confocal scanning across the microfluidic channel for pure αS fibrils. The profile does hardly broaden, as expected for a large amyloid fibril. **f** Diffusion profile for confocal scanning across the microfluidic channel after a single disaggregation round. This leads to significant broadening, whilst the burst height remains similar, indicating that fibrils of similar length and a small species are generated. **g** Comparison of the signal of pure fibrils (blue), single round disaggregation (red) and pure monomer (black). The red signal after a single disaggregation round is the overlay between the two sets.

To further substantiate the above findings, the outcome of a single round of chaperone-mediated disaggregation was measured and the products characterised. For this purpose, αS fibrils were incubated with DnaJB1, Hsc70, and the ATP regeneration system for 5 min to ensure chaperone binding to the fibrils. A single round of the disaggregation reaction was then triggered by the addition of Apg2 and a 200-fold molar excess of the Hsc70-binding peptide GSGNRLLLTG^29–31^ was added to the primed Hsc70–fibril complex (Fig. 4a). Due to the presence of Hsc70-binding peptide, Hsc70 is unable to re-bind to αS fibrils. Note that no disaggregation occurs until Apg2 is added, which results in the release of Hsc70 from fibrils^19^ (see Fig. 4a). This assay thus allowed us to monitor the outcome of only a single round of disaggregation per Hsc70 molecule bound to the fibrils. As shown in Fig. 4b, two αS species were observed: a smaller species with a radius of *R*_h_ = 2.05 ± 0.44 nm, corresponding to monomer, and a larger species with a radius of *R*_h_ = 71.2 ± 3.7 nm, corresponding to the fibril size (cf. *R*_h_ = 74.4 ± 1.0 nm) prior to disaggregation. Due to the conserved fibril length, these data further support the notion that monomer is taken off the fibril ends. In the case of a fragmentation mechanism, in contrast, fibrillar species of smaller length would be observed. As expected, the addition of the Hsc70-binding peptide to an ongoing disaggregation stopped the disaggregation reaction immediately (Supplementary Fig. 11a), and no disaggregation was observed upon addition of peptide before adding Hsc70 to fibrils (Supplementary Fig. 11b). The same behaviour was found when the reaction was carried out with equimolar concentrations of ATP relative to Hsc70 and an excess of the slowly hydrolysable ATP analogue ATP-γ-S (Supplementary Fig. 11c).

Interestingly, the fraction of monomers generated during a single round of disaggregation increased with shorter fibril lengths under constant Hsc70 concentrations (Fig. 4c). As the total αS and Hsc70 concentrations were both 2 µM, there is an excess of chaperone relative to the number of fibrils ends (Supplementary Fig. 11). We investigated, therefore, the extent to which the number of fibril ends governs the rate of disaggregation. In this experiment, the fibril length was decreased by a factor of two to three by sonication, but the total mass concentration of αS was kept constant, resulting in an increased concentration of fibril ends for removal of monomer units. Noteworthily, the fibril population is very heterogeneous (see above, Supplementary Fig. 1c) and we report here on the mean size. The fraction of αS released in a single disaggregation round decreased with the concentration of fibril ends, this effect levelled off for fibril lengths above 100 nm (Fig. 4c), indicative of a critical ratio between Hsc70 concentration and the number of fibril ends for effective disaggregation. To validate this hypothesis, single round experiments were performed with fibrils of identical length distributions but with varying concentrations of the Hsc70 chaperone (Fig. 4d). This experiment revealed that, at an Hsc70 concentration below 1 µM, no disaggregation is observable. This result is interesting, as it suggests a cooperative action or clustering of multiple Hsc70 molecules at individual fibril ends, consistent with a recent report^19^. In contrast, there was no significant difference in the monomer fraction released from fibrils at higher Hsc70 concentrations, suggesting that the number of Hsc70 molecules that can bind to fibril ends is limited (Fig. 4d).

Next, we monitored the outcome of a single round of disaggregation using confocal-based diffusional sizing^48,49^. We first performed experiments on pure fibrils (i.e., in the absence of the disaggregation machinery) (Fig. 4e). As described above, diffusion profiles showed large bursts located at the centre of the channel and profile shapes that do hardly broaden along the channel, as expected for large amyloid fibrils containing numerous labelled monomer units. Fitting of the profile from the confocal measurements yielded a hydrodynamic radius of *R*_h_ = 156.82 ± 3.57 nm consistent with the diffusional sizing experiments in epifluorescence mode and the expected size of fibrils. Strikingly, after a single round of disaggregation, a diffused-out monomeric population with a hydrodynamic radius *R*_h_ = 2.96 ± 0.01 nm appeared in addition to the fibril bursts located at the centre of the channel; the hydrodynamic radius of *R*_h_ = 155.28 ± 1.5 nm of the fibril bursts was conserved compared to the fibril-only experiment. Comparing the diffusion profile of the monomer population obtained in the single round disaggregation experiment with a concentration series performed on labelled αS monomer (Supplementary Fig. 12) revealed that ~100 nM of monomeric protein is present after the single disaggregation round. This suggests that ~5% of the αS population is monomeric after a single round of disaggregation. Importantly, no intermediate species between monomer and fibrils were observed. Together, these findings support the conclusion that the monomer is taken directly off the fibril ends. Assuming an average length fibril consists of 500 monomer units, ~12 monomers would be released per fibril end per disaggregation round.

### Interactions between chaperones and their binding to fibrils

We next focused on the interactions between chaperones as well as between chaperones and fibrils in order to gain further mechanistic insights into the disaggregation reaction. Upon binding of a labelled chaperone to an unlabelled chaperone or fibril, a decrease in its molecular diffusivity concomitant with an increase in its effective molecular weight and size upon binding is expected, thereby allowing us to determine the binding affinity of the respective interactions^16^.

**Fig. 5:**
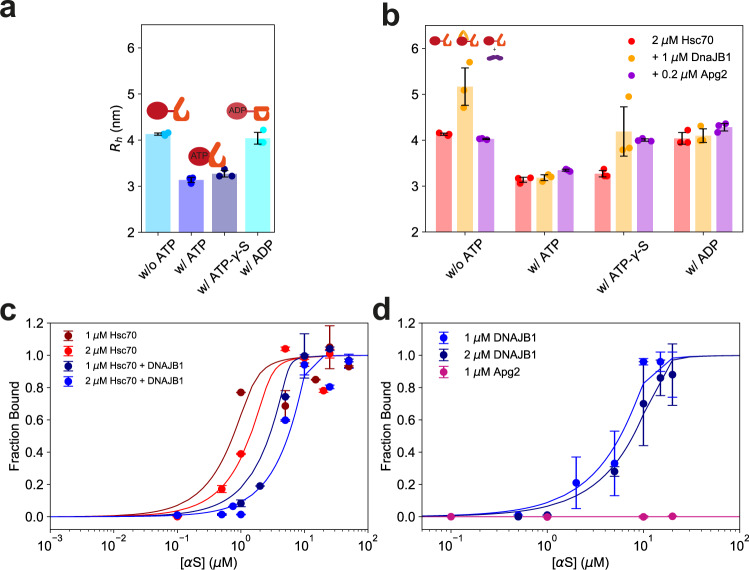
Characterisation of the binding interaction between chaperones and αS fibrils. **a** Conformational compaction of fluorescently labelled Hsc70 upon binding of ATP and ATPγS, as shown by the change in hydrodynamic radius. Binding to ADP preserves the more expanded state. **b** Binding interaction between fluorescently labelled Hsc70 and its co-chaperones. Binding was observed between Hsc70 and DnaJB1, leading to an increase in hydrodynamic radius. DnaJB1 can bind in absence of ATP and in presence of ATP-γ-S but dissociates from Hsc70 in the presence of ATP due to the fast hydrolysis of ATP. **c** Binding of Hsc70 to αS fibrils. Hsc70 shows a binding affinity of *K*_d_ = 139.0 ± 27.6 nM in the presence of ATP (*R*^2^ = 0.98) and a tighter affinity *K*_d_ = 47.5 ± 16.0 nM in the presence of DnaJB1 (*R*^2^ = 0.96), which triggers hydrolysis of ATP to ADP, thereby leading to lid closure and stronger binding. **d** Binding of DnaJB1 and Apg2 to αS fibrils. Apg2 did not bind to αS fibrils. DnaJB1 bound to αS fibrils with an affinity of *K*_d_ = 246.1 ± 28.1 nM (*R*^2^ = 0.98). The concentrations of αS are given with respect to monomer equivalents. Data in **b**–**d** are represented as mean ± standard deviation of *n* = 3 independent experiments.

First, we performed experiments involving Hsc70 in the presence of ATP, ADP, and the non-hydrolysable ATP analogue ATP-γ-S. Note that Hsc70 labelled with Alexa Fluor 647 shows normal disaggregation activity (Supplementary Fig. 2). As shown in Fig. 5a, Hsc70 did undergo a conformational change upon binding of ATP or ATP-γ-S, as reflected in a decrease in its hydrodynamic radius. Conversely, Hsc70 in the ADP bound state has a conformation similar to apo-Hsc70. These observations are consistent with structural analyses of Hsp70 showing that in the ATP state the hydrophobic interdomain linker and the α-helical lid of the SBD are associated with the NBD, and the SBD is in an open conformation, whereas the SBD and NBD are loosely associated in the ADP state^51–55^.

Next, we performed sizing experiments of Hsc70 in the presence of DnaJB1 or Apg2 (Fig. 5b). Hsc70 bound to DnaJB1 in the absence of ATP (*K*_d_ = 46.0 ± 13.5 nM) (Supplementary Fig. 13a), but not in the presence of ATP or ADP; however, binding was detectable in presence of ATP-γ-S. DnaJB1 is known to strongly accelerate the hydrolysis of ATP by Hsc70 and is likely to dissociate from ADP-bound Hsc70^12^. This is consistent with the results obtained here. Evidently, the ATP-state of Hsc70 is short-lived in the presence of DnaJB1, precluding detection of binding in the fluidics system. However, a significant size increase of Hsc70 due to DnaJB1 binding was observed in the presence of the non-hydrolysable ATP analogue ATP-γ-S, which prolongs the ATP-state of Hsc70. Similarly, Hsc70 showed only weak binding to Apg2, consistent with a transient interaction during nucleotide exchange. The size of Apg2 was large (*R*_h_ = 4.46 ± 0.20 nm) (Supplementary Fig. 13b), which may contribute to its effectiveness in the disaggregation reaction in an entropic pulling mechanism^37–39^.

We next investigated the binding between Hsc70 and fibrils. As shown in Fig. 5c in presence of hydrolysable ATP, a binding affinity of *K*_d_ = 139.0 ± 27.6 nM is observed, which is increased to *K*_d_ = 47.5 ± 16.0 nM in the presence of DnaJB1, consistent with DnaJB1 accelerating the hydrolysis of ATP to ADP. Furthermore, stoichiometry analysis of Hsc70 binding to fibrils yielded one Hsc70 molecule per 5.3 ± 0.6 αS monomer units. Notably, as this stoichiometric ratio represents an average value, it is also consistent with the clustering of Hsc70 at fewer binding sites on the fibrils. Indeed, given that we observed no disaggregation at decreased Hsc70 concentrations (see above), our results support the view that clustering of Hsc70 at fibril ends is a critical feature of the disaggregation mechanism. This proposed mechanism is consistent with findings by Wentink et al.^19^, who showed that Hsc70 clustered on αS fibrils when recruited by DnaJB1. Such clustering may also prevent the re-attachment of monomeric units to fibrils, as discussed above (Fig. 2g).

While no binding of Apg2 alone to the fibrils was detectable, DnaJB1 bound the fibrils with an affinity of *K*_d_ = 246.1 ± 28.1 nM (Fig. 5d), consistent with the role of DnaJB1 in recruiting Hsc70.

## Discussion

Hsc70 cooperates with its co-chaperones DnaJB1 and Apg2 (Hsp110) in disaggregating αS fibrils^18^. By bringing together microfluidic measurements with chemical kinetics and thermodynamic analysis, we have investigated this process here in a quantitative manner. We have found that, during the disaggregation reaction, only two species are significantly populated, namely larger, fibrillar species and αS monomer units released from the fibrils. The time-dependent size decrease of the larger species is consistent with pseudo-first-order kinetics, under conditions where Hsc70 is in excess and the concentration of fibrils is limiting. Thus, αS monomers are taken off the fibrils directly. Indeed, the pure monomer is abundant almost immediately after the addition of the chaperones, and the monomer fraction increases linearly. An advantage of microfluidic diffusional sizing is that the end product of the disaggregation reaction, monomeric αS, can be detected, which is not ThT active and thus not observable in traditional disaggregation assays. Interestingly, our results indicate that each disaggregation event is independent of the initial fibril lengths, while the rate is slightly accelerated for shorter fibrils as shown with a change in the rate constant with respect to the total concentration of synuclein. As a single round of chaperone action produces only monomer and fibrils, this further suggests that the monomer is taken off the fibril ends. Dissociation of monomers from within the fibrils would have resulted in a substantial decrease in fibril length due to induced fragmentation. Future directions will include investigating the effect of different fibril polymorphs and αS variants on the disaggregation kinetics.

**Fig. 6:**
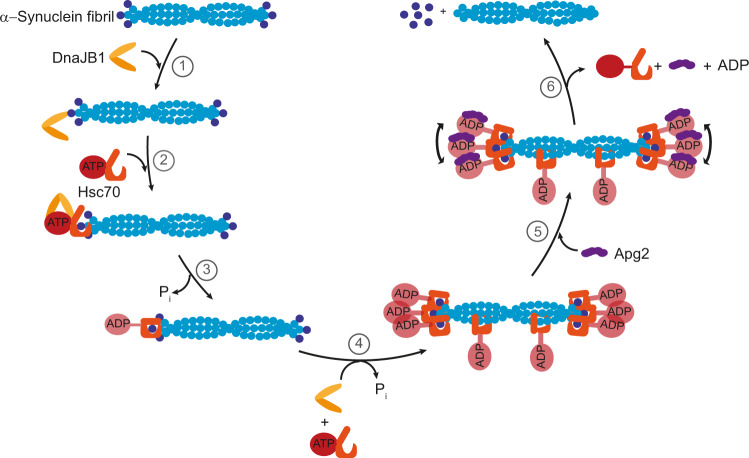
Emerging principles of Hsc70 disaggregase activity. Model for Hsc70-mediated protein disaggregation of αS fibrils. This disaggregation mechanism comprises six steps: step 1, DnaJB1 binding to αS fibrils; step 2, Hsc70 loading onto fibrils mediated by DnaJB1; step 3, hydrolysis of Hsc70-bound ATP to ADP and dissociation of DNAJB1; step 4: further Hsc70 loading resulting in clustering of Hsc70 at fibril ends; step 5, Apg2 binding to Hsc70; step 6, dissociation of αS monomer coupled to ADP dissociation from Hsc70 and disassembly of the chaperone machinery.

Our analysis of the disaggregation kinetics and the interactions between the chaperone components and their interactions with αS fibrils, together with the previous reports^7,19^, leads to a picture of chaperone function as shown in Fig. 6 and suggests that the disaggregation reaction can be divided into the following six steps: DnaJB1 binds first to the fibrils (step 1) and recruits Hsc70 in the ATP state. ATP hydrolysis on Hsc70, accelerated by DnaJB1, results in tight binding of Hsc70 to fibrils (step 2), subsequent hydrolysis of ATP to ADP (step 3), and clustering of Hsc70 on the fibril ends (step 4, see below). Disaggregation resulting in αS monomer production then occurs upon addition of Apg2 (step 5/6). As Apg2 alone does not detectably interact with the fibrils, it may act solely as a NEF, although interaction with the fibril substrate in the presence of Hsc70 cannot be ruled out. The large size of Apg2 may facilitate disaggregation according to the model of entropic pulling^37–39^, consistent with recent findings that the larger size of Apg2 relative to other NEFs of Hsc70 plays a role^19^. Our data suggest further that disaggregation requires clustering of Hsc70 molecules, with repulsive forces between αS-bound Hsc70 molecules inducing monomer dissociation from fibril ends (step 5). This could be mechanistically similar to the observed conformational expansion of non-native proteins by the binding of multiple Hsp70 to sites within the same polypeptide chain^31,56^. Alternatively, since αS monomer dissociation from the fibril critically depends on Apg2, we speculate that binding of Apg2 to clustered Hsc70 molecules induces the steric repulsion that drives disaggregation (Fig. 6). Moreover, clustering likely functions to prevent re-attachment of monomeric units to fibrils. However, given the stoichiometry of approximately one Hsc70 bound per 6 αS monomers, it is likely that Hsc70 binds along the fibril surface as well, and that only binding to the fibril ends is productive. Binding along the fibril surface may be involved in reducing secondary nucleation of aggregation by αS monomers^1,57^. This unproductive binding and the necessity for having clustered chaperones explains why, despite nanomolar affinity, no disaggregation occurs below a Hsc70 concentration of 1 μM, as shown in Fig. 4d. Further directions to extend on our studies should include investigation of these phenomena with various other substrates, including the amyloid-β peptide or Tau protein, key players in the onset and progression of Alzheimer’s disease.

In conclusion, we present here a comprehensive kinetic and thermodynamic description of a complex protein quality control mechanism allowing the clearance of amyloidogenic deposits of the protein αS. We found that, in the disaggregation reaction of αS fibrils, the monomer is taken directly from the fibril ends, apparently mediated by the Hsc70 chaperone system assembling in clusters at the fibril ends. This process follows single exponential kinetics and is highly effective, such that it leads to complete disaggregation of amyloid fibrils, thereby likely contributing to the prevention of Parkinson’s disease.

## Methods

### Materials

All chemicals were purchased in the highest purity available. Hydroxyethyl piperazineethanesulfonic acid (HEPES), potassium hydroxide (KOH), potassium chloride (KCl), dithiothreitol (DTT), and Tween20 were purchased from Sigma Aldrich (St. Louis, MO, USA) and were of analytical grade. Pyruvate kinase (10109045001) from rabbit muscle was purchased from Roche (Basel, Switzerland). Poly-(dimethylsiloxane) (PDMS) and curing agent were purchased from Momentive (Techsil, Bidford-on-Avon, UK) and carbon nanoparticles (Plasmachem, Berlin, Germany) were added as previously described^41^. For all microfluidic experiments, the buffer used contained 50 mM HEPES–KOH (pH 7.5), 50 mM KCl, 5 mM MgCl_2_, 2 mM DTT; co-flow buffer was additionally supplied with 0.01% Tween20.

### Expression and purification of molecular chaperones and αS monomer

Recombinant human wildtype αS was purified as described previously^58^. In brief, *Escherichia coli* BL21(DE3) cells were transformed with pT7-7 αS and cultured in a lysogenic broth (LB) medium. Protein expression was induced by 1 mM isopropyl β-d-1-thiogalactopyranoside (IPTG) for 4 h at 37 °C. Bacteria were harvested, and pellets were lysed in 10 mM Tris–HCl (pH 8.0), 1 mM EDTA, 1 mM phenylmethylsulfonyl fluoride (PMSF). The lysate was sonicated for 5 min and boiled subsequently for 15 min, followed by centrifugation. The supernatant was subjected to streptomycin sulfate and ammonium sulfate precipitation steps as described. The ammonium sulfate pellet formed after centrifugation at 5200×*g* for 30 min was dissolved in 50 mM Tris–HCl (pH 7.5), 150 mM KCl and subjected to size exclusion chromatography (SEC) on a Superdex 200 column (GE Healthcare, Chalfont St Giles, UK). αS fibrils were generated by shaking a mixture containing 10% labelled and 90% unlabelled monomer at 37 °C and 200 rpm for 4 days. Fibrils were sonicated (cycle 0.3, power 10%, 90 s) using a Sonopuls ultrasonic homogeniser (Bandelin, Nänikon, Switzerland).

Human Hsc70, DnaJB1, and Apg2 were expressed in *E. coli* BL21(DE3) as fusion proteins with protease-cleavable His_6_- or His_6_-Smt3 tags and purified by tandem Ni-affinity chromatography with intermittent protease cleavage similar as described previously^59^.

Hsc70 was expressed from the plasmid pProEx-HtA Hsc70. Cells were grown in LB medium at 37 °C to an OD_600_ ∼0.5 and induced with 0.5 mM IPTG for 18 h at 21 °C. Cells were lysed by ultrasonication in 50 mM HEPES–KOH (pH 8.0), 10 mM KCl, 5 mM MgCl_2_ (buffer A) containing 0.8 mg/mL lysozyme at 4 °C. The supernatant after centrifugation at 125,000×*g* for 45 min was applied to a Ni-NTA column (GE Healthcare, Chalfont St Giles, UK) equilibrated in buffer A. The column was washed with a step gradient of buffer A containing increasing amounts of imidazole (20/250/1000 mM). The bound protein was eluted with buffer A containing 250 mM imidazole. This was followed by cleavage of the His_6_-moiety at 4 °C with His_6_-tobacco etch virus (TEV) protease for 45 h. After transfer into buffer A using a desalting column, the material was passed over the Ni-NTA column and the flow-through was collected. Next, Hsc70 was purified by anion exchange chromatography on MonoQ (GE Healthcare, UK) in the same buffer system using a linear salt gradient (0–700 mM KCl) in 50 mM HEPES–KOH (pH 8.0), 5 mM MgCl_2_. Finally, the Hsc70-containing fractions were subjected to SEC on Superdex 200 column (GE Healthcare, Chalfont St Giles, UK) in 50 mM HEPES–KOH (pH 8.0), 150 mM KCl, 5 mM MgCl_2_ and 5% glycerol.

DnaJB1 and Apg2 were expressed from the plasmids pCA528-DnaJB1 and pCA528-HspA4, respectively^17^. Cells were grown in LB medium at 37 °C to an OD_600_ ∼0.5 and induced with 0.5 mM IPTG for 5.5 h at 30 °C. Cells were lysed with an Emulsiflex (Avestin, Ottawa, Canada) cell disruptor in 50 mM HEPES–KOH (pH 7.4), 10 mM KCl, 5 mM MgCl_2_ (buffer B) containing 2 mM PMSF and Complete protease inhibitor cocktail (Roche). After centrifugation, the supernatant was applied to a Ni-NTA column equilibrated in buffer B. After washing with buffer B, the bound protein was eluted with buffer B containing 250 mM imidazole. The His_6_-Smt3 moiety was cleaved with PEN2 protease (MPIB Core facility) at 4 °C in the presence of 1 mM dithiothreitol (DTT). After buffer exchange, the mixture was passed over the Ni-NTA column and the flow-through was collected. SEC on Sephacryl S-200 (GE Healthcare, UK) in 50 mM Tris–HCl (pH 8.0), 5 mM MgCl_2_ and 150 mM KCl (buffer C) was the final purification step for Apg2. DnaJB1 was further purified by SEC on Sephacryl S-100 in buffer C containing 5% glycerol, and by cation exchange chromatography on Source 30S (GE Healthcare), wherein the elution was carried out with a linear salt gradient (0–400 mM NaCl) in 50 mM Tris–HCl (pH 7.5).

Hsc70, DnaJB1, and Apg2 were buffer exchanged to 0.1 M NaHCO_3_ and incubated with 3 molar equivalents of Alexa Fluor 647 overnight at 4 °C. Free dye was removed by size exclusion chromatography on a Superdex 200 increase column with 50 mM HEPES–KOH, pH 7.5, as elution buffer, yielding labelled chaperones with 1.7 labels per molecule.

### Microfluidic diffusional sizing

A scheme of the chip design is shown in Fig. 1. Fabrication and operation of the microfluidic devices for microfluidic diffusional sizing have been described previously^40,60^. Briefly, the microfluidic devices were fabricated in PDMS by standard soft-lithography techniques and bonded onto a glass coverslip after activation with oxygen plasma. Sample loading from reservoirs connected to the respective inlets and control of flow rate was achieved by applying negative pressure at the outlet using a glass syringe (Hamilton, Bonaduz, Switzerland) and a syringe pump (neMESYS, Cetoni GmbH, Korbussen, Germany). A custom-built inverted epifluorescence microscope equipped with a charge-coupled-device camera (Prime 95B, Photometrics, Tucson, AZ, USA) and brightfield LED light sources (Thorlabs, Newton, NJ, USA) was used to record the images, using the Cy5-4040C-000 Filter set from Semrock (Laser 2000, Huntingdon, UK) for detection of Alexa647-labelld chaperones and a fluorescent filter set with an excitation filter at 475 ± 35 nm, emission filter at 525 ± 30 nm and dichroic mirror for 506 nm (Laser 2000, Huntingdon, UK) for detection of Alexa-488 labelled αS. Images were taken using Micro Manager (Version l.4.23 20170327), typically at flow rates 20, 60, and 100 μL/h, and lateral diffusion profiles were recorded at four different positions along the microfluidic channels.

Diffusional sizing experiments involving confocal microscopy were done on a custom-built laser confocal microscopy setup. Briefly, the microscope is equipped with a 488-nm laser line (Cobolt 06-MLD, Hübner Photonics, Derby, UK) and a single-photon counting avalanche photodiode (SPCM-14, PerkinElmer, Seer Green, UK) for subsequent detection of emitted fluorescence photons. Further details of the optical unit have been described previously^48,49^. Diffusion profile recording was done by continuously moving the confocal observation volume through the centre four channels of the microfluidic device. Profiles were typically taken at a flow rate of 100 μL/h, and lateral diffusion profiles were recorded at four different positions along the microfluidic channels.

Diffusion profiles extracted from fluorescence images and confocal recordings were fitted using a custom-written analysis software by numerical model simulations solving the diffusion–advection equations for mass transport under flow^42^. For evaluation of the disaggregation time courses, we assumed two species representing the fibrils and the dissociated monomers. For the thermodynamic evaluation and sizing of pure species, we fitted the diffusion profiles with one species only to determine the average size of the bound and unbound chaperone.

### Kinetic measurements

The reaction mixture for disaggregation measurements contained 2 μM αS fibrils, 2 μM Hsc70, 1 μM DnaJB1, 0.2 μM Apg2, 5 mM 2-phosphoenolpyruvate, 0.05 mg/mL pyruvate kinase, and 5 mM ATP in 50 mM HEPES–KOH (pH 7.5), 50 mM KCl, 5 mM MgCl_2_, 2 mM DTT. This mixture was incubated at 300 rpm and 30 °C. At different time points, 8 µL aliquots were taken out, injected into the microfluidic chip and images were acquired as described above. The rate of monomer release is proportional to the number of ends in the system. As shorter fibrils are consumed, the number of ends decreases. We thus approximate the time evolution of the mass concentration, *M,* of fibrils as:

1a $$\frac{{\rm {d}}M}{{\rm {d}}t}=-{k}\cdot M(t)$$

1b $$M(t)=M({t}_{0}){{\rm {e}}}^{-kt}$$

Therefore, the hydrodynamic radius of the fibril population at time *t* is described by:

2 $${R}_{{\rm {h}}}(t)={R}_{{\rm {h}}}({t}_{0}){{\rm {e}}}^{-kt}$$

Likewise, the concentration of monomer, *m*, increases as

3 $$\frac{{\rm {d}}M}{{\rm {d}}t}=-\frac{{\rm {d}}m}{{\rm {d}}t}$$

or, in its integrated and normalised form, the fraction of monomer, *f*_m,_ becomes

4 $${f}_{{{{{{\rm{m}}}}}}}(t)=(1-{{\rm {e}}}^{-kt})$$

### Single round disaggregation experiments

The reaction mixture for disaggregation measurements contained 2 µM αS fibrils, 2 µM Hsc70, 1 µM DnaJB1, 5 mM 2-phosphoenolpyruvate, 0.05 mg/mL pyruvate kinase, and 5 mM ATP in 50 mM HEPES–KOH (pH 7.5), 50 mM KCl, 5 mM MgCl_2_, 2 mM DTT. This mixture was incubated at 300 rpm and 30 °C for 5 min. Subsequently, 0.2 µM Apg2 and 400 µM Hsc70-binding peptide GSGNRLLLTG^29–31^ were added. This peptide can bind to Hsc70, thereby preventing substrate rebinding and, thus, terminating the disaggregation.

### Thermodynamic characterisation

For binding experiments, samples were prepared in typically 30 μL total volume, using the same working concentrations of the interacting partners considered as in the disaggregation time course described above under the same buffer conditions. The protocol for the equilibrium binding curves was adapted from the previous reports^16^. The concentration of one of the interacting molecules was varied between 0.1 and 10 µM accordingly, while the labelled component was held at a constant concentration equal to the working concentration discussed previously. The samples were typically incubated for 30 min and then measured in triplicates in three independent channels at three flow rates.

### Atomic force microscopy

AFM was performed on positively functionalized mica substrates.10 μL of 0.5% (v/v) 3-aminopropyl-triethoxysilane (APTES, Sigma) in Milli-Q water was deposited onto freshly cleaved mica and incubated for 1 min. The substrate was rinsed three times with 1 mL of Milli-Q water and dried by a gentle stream of nitrogen gas. Finally, for each sample, an aliquot of 10 μL of the solution was deposited on the positively functionalized surface. The droplet was incubated for 5 min, then rinsed with 1 mL of Milli-Q water and dried under nitrogen gas. The preparation was carried out at room temperature. AFM maps were acquired using an NX10 AFM (Park Systems) operating in non-contact mode and equipped with a silicon tip (PPP-NCHR, 42 N/m) with a nominal radius <10 nm. Image flattening was performed by SPIP (Image Metrology) software.

### Reporting summary

Further information on experimental design is available in the Nature Research Reporting Summary linked to this paper.

## Supplementary information

Supplementary Information

Reporting summary

## Data Availability

The raw data and analysis code underlying this study will be made available upon reasonable request. Source data are provided with this paper. All data generated in this study and included in this manuscript have been deposited in the Figshare database under 10.6084/m9.figshare.15173088. Source data are provided with this paper.

## Source data

Source Data

## Code availability

Code available under https://zenodo.org/record/3881940#.YRGzCSV4UlQ.
