# Supplementary material for: The Hsc70 disaggregation machinery removes monomer units directly from α-synuclein fibril ends
Source: Nat Commun. 2021 Oct 14;12:5999. doi: 10.1038/s41467-021-25966-w (PMC8516981; doi:10.1038/s41467-021-25966-w)
Supplement: Supplementary file 1 — Supplementary Information [file 41467_2021_25966_MOESM1_ESM.pdf]

The Hsc70 Disaggregation Machinery Removes Monomer Units Directly from α-Synuclein Fibril Ends

Supplementary Information

Matthias M. Schneider[1,+], Saurabh Gautam[2,3,+], Therese W. Herling[1,+], Ewa Andrzejewska[1,+], Georg Krainer[1,+], Alyssa Miller[1], Victoria Trinkaus[2], Quentin A. E. Peter[1], Simone Francesco Ruggeri[1], Michele Vendruscolo[1], Andreas Bracher[2], Christopher M. Dobson[1,†], F. Ulrich Hartl[2,*], Tuomas P. J. Knowles[1,4,*]

[1] Centre for Misfolding Diseases, Yusuf Hamied Department of Chemistry, University of Cambridge, Lensfield Road, Cambridge CB2 1EW, United Kingdom

[2] Department of Cellular Biochemistry, Max-Planck Institute of Biochemistry, Am Klopferspitz 18, 82152 Martinsried, Germany

[3] Present affiliation: ViraTherapeutics GmbH, 6063 Rum, Austria

[4] Cavendish Laboratory, Department of Physics, University of Cambridge, JJ Thomson Road, Cambridge CB3 0HE, United Kingdom

[+] These authors contributed equally

[†] Deceased (September 2019)

[*] To whom correspondence should be addressed: uhartl@biochem.mpg.de (FUH), tpjk2@cam.ac.uk (TPJK)

Supplementary Figures

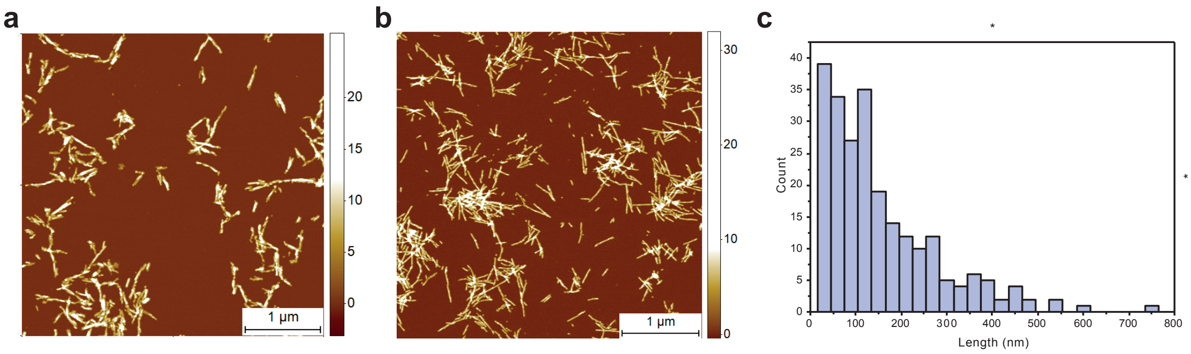

Supplementary Figure 1 : AFM imaging to compare labelled and unlabelled αS fibrils. (**a**) unlabelled and (**b**) labelled fibrils were imaged by atomic force microscopy (AFM). (**c**) A length analysis of labelled fibrils is consistent with diffusional sizing assays. For a-c, 20 scans were taken per sample. The location of imaging was chosen systematically, with the same pre-determined areas of the mica being imaged to ensure comparability between the samples.

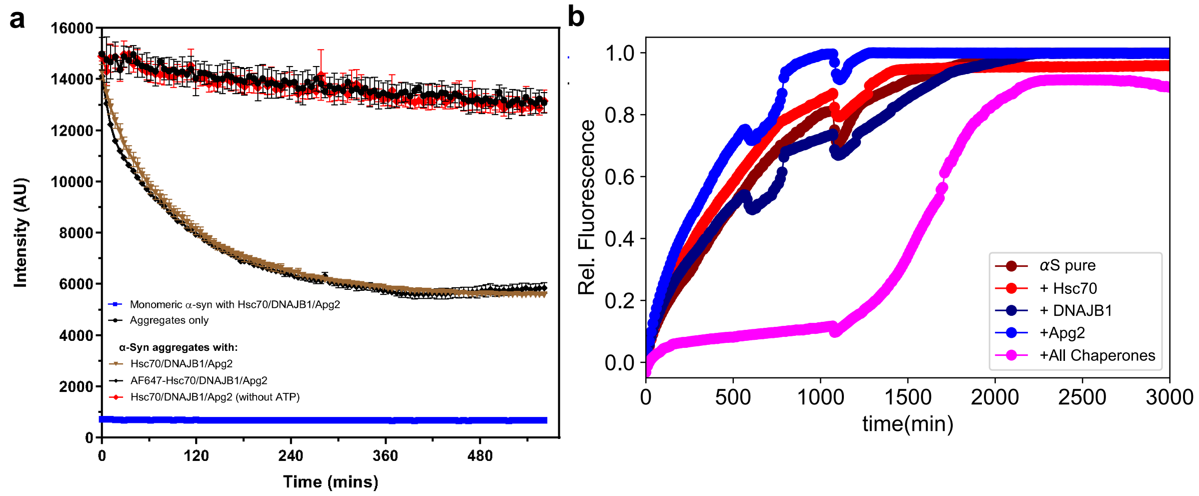

Supplementary Figure 2 : Kinetics of αS aggregation and disaggregation by the Hsc70-DnaJB1-Apg2 system in the presence of ATP. (**a**) Disaggregation was followed by ThT fluorescence. Protein concentrations were: Hsc70 2 μM, DnaJB1 1 μM; Apg2 0.2 μM, ATP 5 mM. ThT fluorescence is decreasing over time when all chaperones are present. Consistent results when Alexa Fluor 647 labelled Hsc70 was used. (**b**) Aggregation of αS in HEPES-KOH, pH 7.5, under heavily seeded conditions (300 muM at 30 muM αS conditions, i.e. 1% of the mass concentration are seeds to follow elongation kinetics.

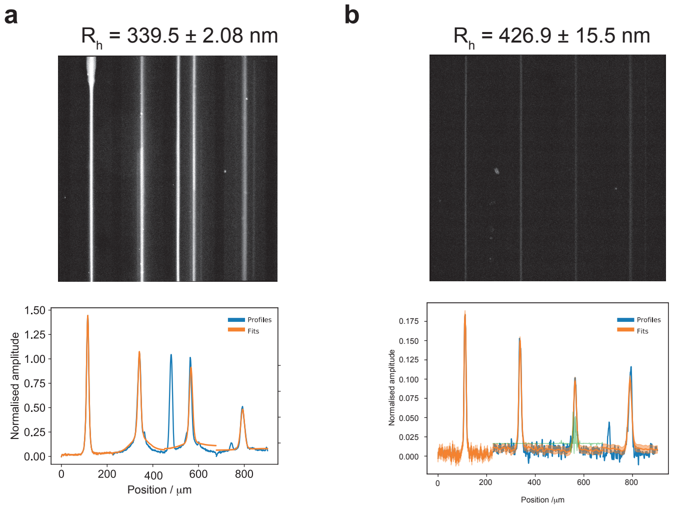

Supplementary Figure 3 : Diffusional Sizing of pure fibrils at different lengths. (**a**) 340 ± 2 nm and (**b**) 427 ± 16 nm.

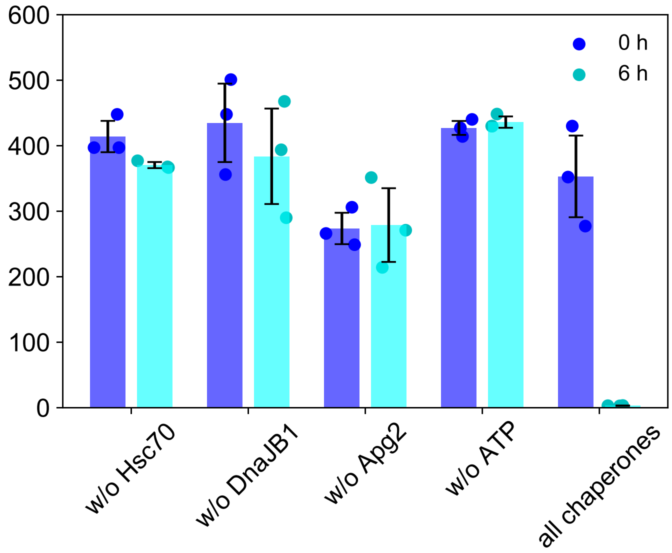

Supplementary Figure 4 : Negative Controls. Disaggregation by MDS in absence of the chaperone Hsc70 as well as the co-chaperones DnaJB1 and Apg2, or in the absence of ATP at 0 h (blue) and 6 h (cyan). No decrease in hydrodynamic radius, R_h, is observable after 6 hours, while a size decrease is observable in presence of all chaperones. Error bars represent the standard deviation (n = 3 independent measurements).

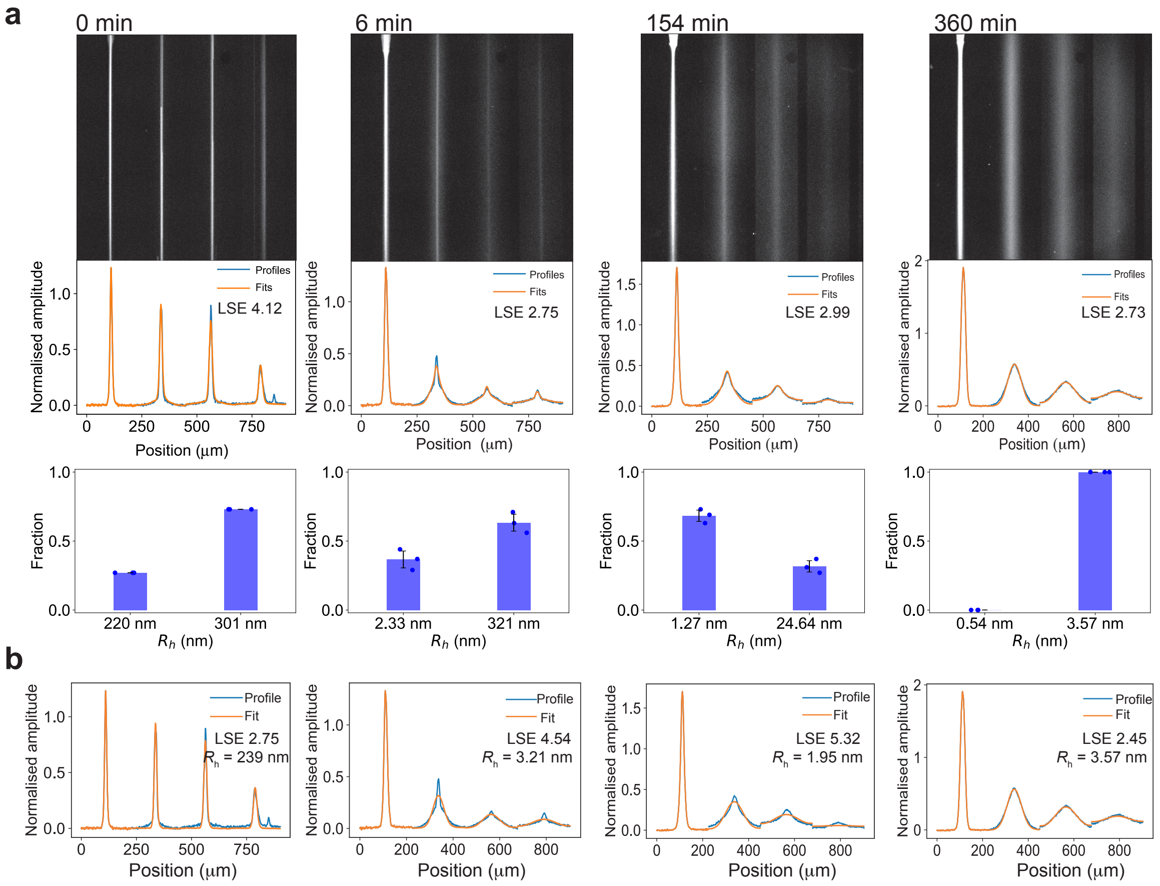

Supplementary Figure 5 : Statistical comparison of one and two species fit in microfluidic image analysis. (**a**) 2 species and (**b**) 1 species of the images shown in Figure 2. The distributions at time points t = 6 min and t = 154 min fit better to two species than to one species, as the reduced least squares estimation (LSE) is significantly lower for 2 species than 1 species. In contrast, the time points t = 0 min and t = 360 min are better represented by 1 species than 2 species, as it would be assumed given that only one species is present. Data in a are represented as mean ± standard deviation of n = 3 independent experiments.The individual data points are overlaid.

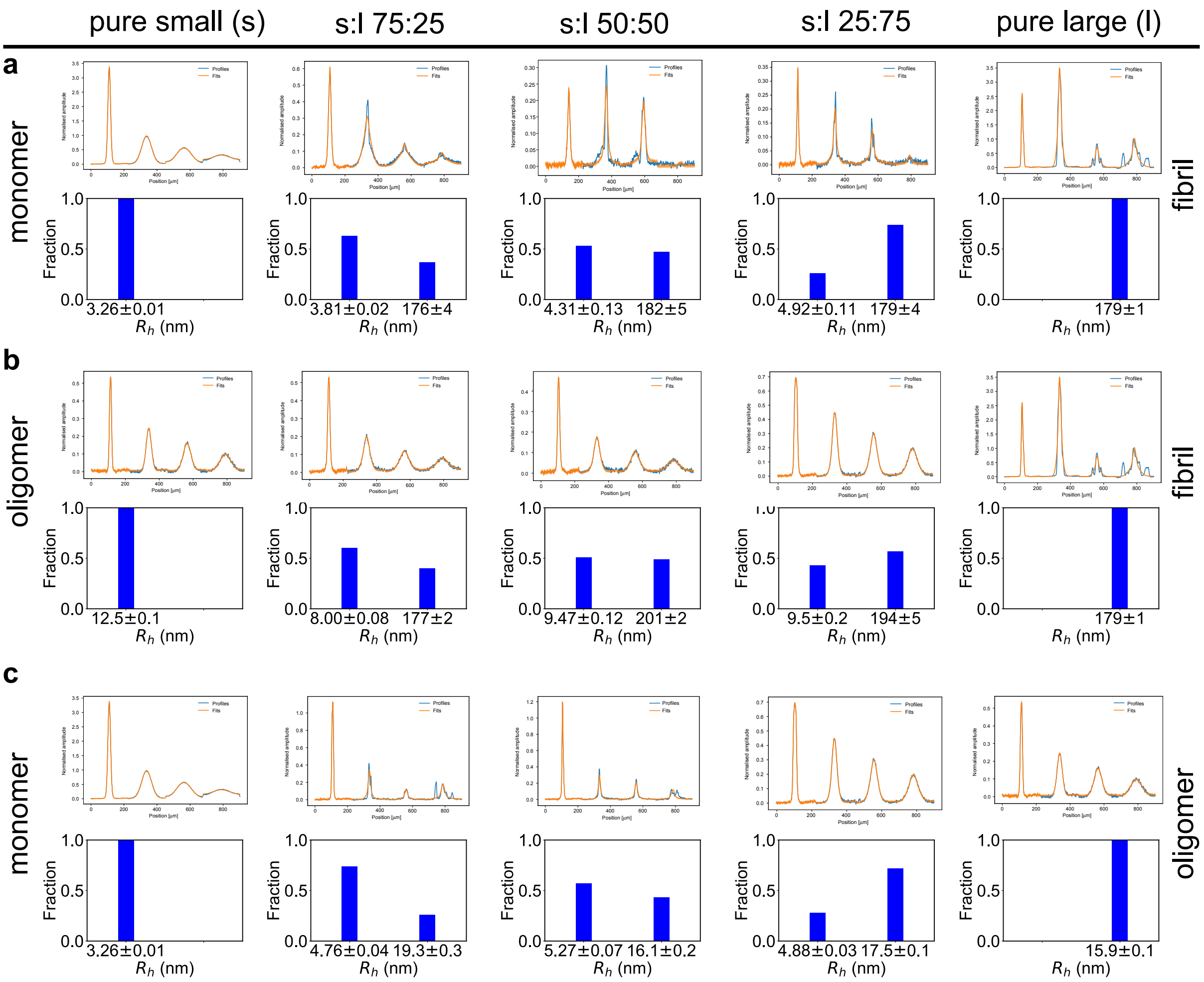

Supplementary Figure 6 : Control experiment demonstrating the ability of microfluidic diffusional sizing (MDS) to distinguish αS fibrils, oligomers and monomers in heterogeneous mixtures. (**a**) Monomer and fibril, (**b**) oligomer and fibril and (**c**) monomer and oligomer at identical mass concentration were mixed in different ratios. The fits are displayed for each mixture in the top row, the bottom row shows the aspect ratio and the radii for both species fitted.

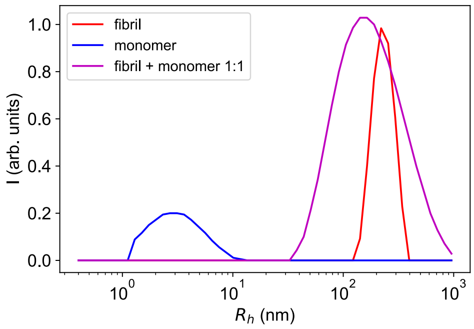

Supplementary Figure 7 : Challenges of dynamic light scattering (DLS) to detect monomers in the presence of fibrils. DLS of monomer, fibrils, and 1:1 mixture of monomer and fibrils, with a total mass concentration of 20 μM αS, revealing a hydrodynamic radius of 269 ± 49 nm for pure fibrils, 250 ± 80 nm for the 1:1 mixture, and 3.3 ± 1.9 nm for pure monomer. Data in a and b are represented as mean ± standard deviation of n = 3 independent experiments.

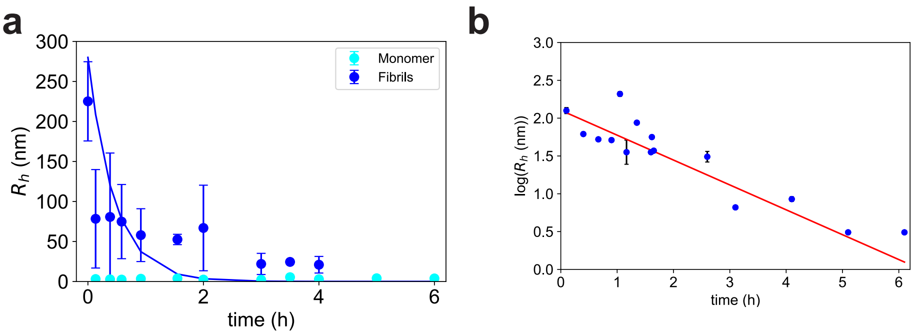

Supplementary Figure 8 : Disaggregation of sonicated αS fibrils. (**a**) Size population of monomers and fibrils over time, starting from 280 nm initial size. The size of the larger species decays with single exponential kinetics (solid line). The size of the smaller species appearing is conserved (Fig. 2e). Error bars represent the standard deviation (n = 3 independent measurements). (**b**) Kinetic fits of log(R_h) vs. time, according to the kinetic model described in equation 1 in materials and methods. From these fits, a rate constant $k = 2.2 \cdot 10^{-4} \pm 0.2 \cdot 10^{-4}$ s^{-1} was determined. $R^2 = 0.87$. Data in a, b and c are represented as mean ± standard deviation of n = 3 independent experiments.

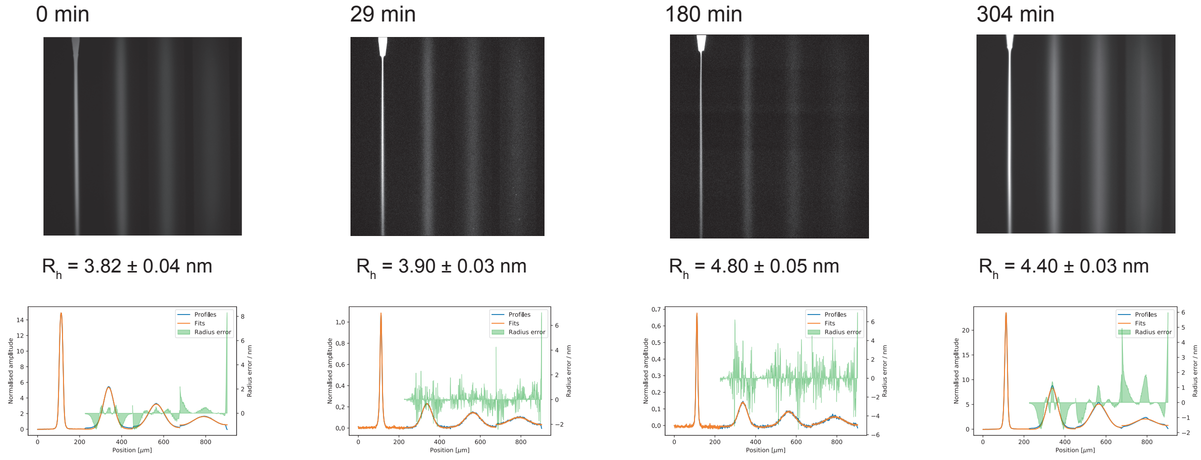

Supplementary Figure 9 : Hydrodynamic radius of Hsc70 at different times during disaggregation. The small size ($R_h \approx 3.9$ nm) indicates that the majority of the Hsc70 remains in an unbound state with only little Hsc70 in the bound state, indicating an excess of Hsc70 chaperone over aS fibrils. Time point at t=0 is pure Hsc70 before mixing with αS fibrils.

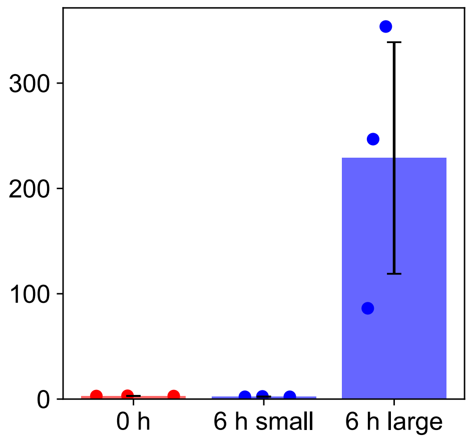

Supplementary Figure 10 : Negative Controls. Addition of labelled monomer to unlabelled fibrils. After 6 h, a large species of $229 \pm 53nm$ is observed as dominant species (90%) along with the monomer, indicating incorporation of monomer in unlabelled fibrils. Data in c, d, e, f and g are represented as mean ± standard deviation of n = 3 independent experiments.The individual data points are overlaid.

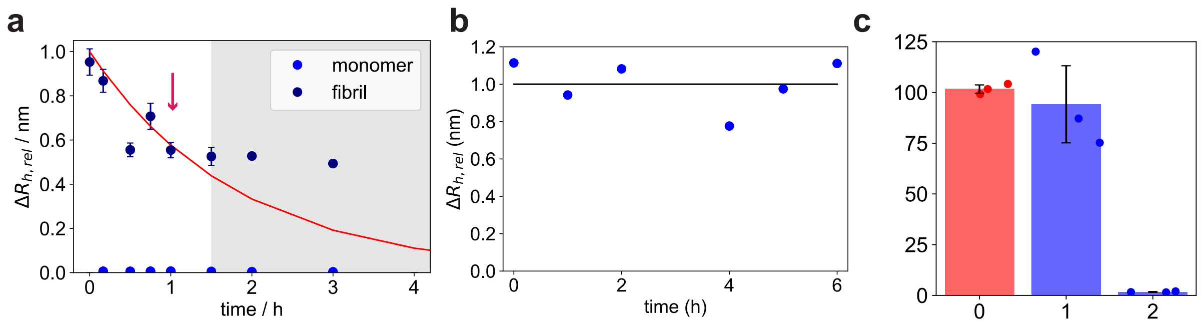

Supplementary Figure 11 : Inhibition/quenching of αS disaggregation by Hsc70 binding peptide and slowly hydrolysable ATP analogue. (**a**) Disaggregation time course was observed for 60 minutes, followed by addition of Hsc70 binding peptide (arrow) and monitoring disaggregation for another 120 minutes. The red line shows the progression of disaggregation in the absence of peptide. The behaviour of aS monomer is also shown. Error bars represent the standard deviation (n = 3 independent measurements). (**b**) Disaggregation time course with addition of Hsc70 binding peptide before Hsc70. The hydrodynamic radius of aS fibrils is conserved over 6 hours, showing that the disaggregation does not proceed in presence of the Hsc70 binding peptide. (**c**) Single Round experiment with the slowly hydrolysable ATP analogue ATP-γ-S, which cannot be dissociated by Apg2 and, therefore, stops the disaggregation after a single round. This shows that a single disaggregation round of fibrils of initial length 101.68 ± 2.51 nm results in the occurrence of two species, a fibrillar species of 94.17 ± 3.96 nm and a monomeric species of 2.11 ± 0.05 nm. Error bars represent the standard deviation (n = 3 independent measurements). The individual data points are overlaid in c.

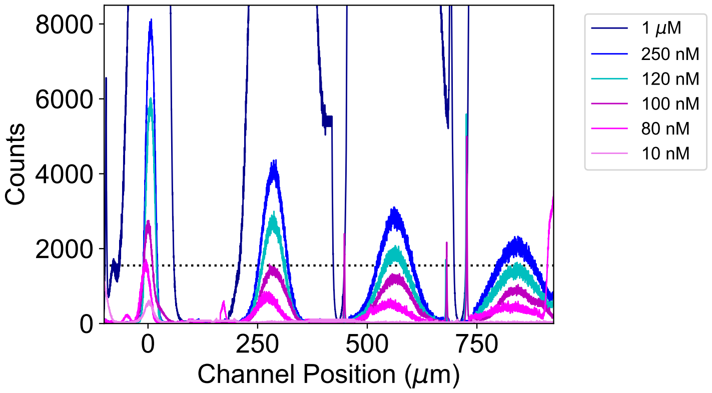

Supplementary Figure 12 : Fluorescence intensity of αS monomer at different concentrations. This data is used as calibration for the data in Fig. 4g.

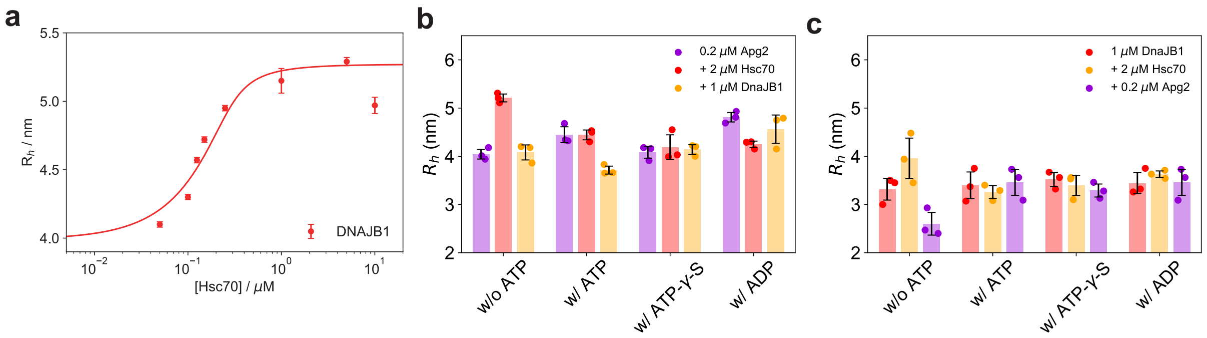

Supplementary Figure 13 : Binding of co-chaperones. (**a**) Binding curve for the interaction between Hsc70 and DnaJB1 (K_d = 46.0 ± 13.5 nM) Error bars represent the standard deviation (n = 3 independent measurements). . Binding of (**b**) labelled Apg2 and (**c**) labelled DnaJB1 to Hsc70 with different ATP/ADP conditions, co-chaperone, yielding results consistent with Fig. 5a-b for labelled Hsc70. Data in a, b and c are represented as mean ± standard deviation of n = 3 independent experiments. The individual data points are overlaid.
